# Machinability and Surface Generation of Pd_40_Ni_10_Cu_30_P_20_ Bulk Metallic Glass in Single-Point Diamond Turning

**DOI:** 10.3390/mi11010004

**Published:** 2019-12-18

**Authors:** Jie Xiong, Hao Wang, Guoqing Zhang, Yanbing Chen, Jiang Ma, Ruodong Mo

**Affiliations:** College of Mechatronics and Control Engineering, Shenzhen University, Nan-hai Ave 3688, Shenzhen 518060, China; xiongjie2017@email.szu.edu.cn (J.X.); whao@szu.edu.cn (H.W.); 1800291004@email.szu.edu.cn (Y.C.); MrDream0426@163.com (R.M.)

**Keywords:** bulk metallic glass, single-point diamond turning, surface quality, molecular dynamics simulation

## Abstract

Pd_40_Ni_10_Cu_30_P_20_ bulk metallic glass (BMG) is widely used in industrial fields due to its excellent oxidation resistance, corrosion resistance, and thermal stability. However, the lack of research on the machinability and cutting performance of BMG using single-point diamond turning (SPDT) limits its application for engineering manufacturing. In the present research, a series of turning experiments were carried out under different cutting parameters, and the machinability reflected by the quality of machined surface, chip morphology, and tool wear were analyzed. Based on the oxidation phenomenon of the machined surface, a molecular dynamics (MD) simulation was conducted to study the mechanism and suppression of the machined surface oxidation during the cutting. The results show that: (1) The Pd-based BMG had good machinability, where the machined surface roughness could go down to 3 nm; (2) irregular micro/nanostructures were found along the tool path on the outer circular region of the machined surface, which greatly affected the surface roughness; and (3) the cutting heat softened the workpiece material and flattened the tool marks under surface tension, which improved the surface quality. This research provides important theoretical and technical support for the application of BMG in optical mold manufacturing.

## 1. Introduction

Bulk metallic glass (BMG), also known as amorphous alloy, has a disordered array of atoms, no crystal grains, grain boundaries and dislocations, uniform microstructure, no precipitation phase, and the same structure as glass [1,2]. BMG has become a new type of engineering material, whose metastability provides unusual material properties, such as excellent strength, hardness and elastic strain limit, and excellent corrosion and wear resistance, as well as unique physical properties, such as thermal, electromagnetic, and electrical properties [3,4,5,6,7].

BMG was first obtained by Klement in 1960 through the rapid quenching method [8]. After that, the research on BMG has developed rapidly, setting off the first climax of condensed matter physics and materials research. BMG has the hardness of ceramics, but when heated to the supercooled liquid state, it softens like plastic and exhibits Newtonian fluid properties. Therefore, it is an ideal micro/nanomachining material [9], which has been widely used in the field of manufacturing and molding. In the field of 3D printing, Shen et al. used 3D-printed Zr_52.5_Ti_5_Al_10_Ni_14.6_Cu_17.9_ BMG to manufacture very complex and highly elastic components [10]. Zhang et al. successfully manufactured gears with excellent mechanical properties, such as a high fracture strength (≈2 GPa) and very good fracture toughness (13–21 MPa·m^1/2^), by using thermal-sprayed 3D printing Fe-based BMG/stainless steel composites [11]. In the field of mold manufacturing, Pan et al. proposed a new preparation process for an aspheric lens using Mg_58_Cu_31_Y_11_ BMG rapid prototyping material as an auxiliary mold, and the hardness of the BMG mold was as high as 3.445 GPa [12]. In the field of coatings, Lee et al. used magnetron sputtering deposition technology to deposit ZrCu BMG on a pet substrate to obtain an ITO/ZrCu double-layer transparent conductive electrode metal layer, making the ZrCu layer form a continuous and smooth film with a thickness of less than 6 nm, such that some special functions could be realized [13]. Chu et al. used BMG with a high elastic limit, corrosion resistance, and friction resistance as a coating material, which not only provided the workpiece with excellent mechanical properties, but also provided better wear and oxidation resistance than ordinary materials [14]. In the field of ultra-precision machining, Fujita et al. studied the cutting characteristics of BMG by using different tool materials, tool nose radius, and cutting velocity. The materials of Zr_65_Cu_15_Ni_10_Al_10_ BMG, Pd_40_Ni_10_Cu_30_P_20_ BMG, steel (JIS SGD-400D), and free-cutting brass (JIS C3604) were selected for the cutting comparison. The experimental results showed that although the tensile strength of BMG was twice that of steel, the main cutting force value of BMG was half of that of steel. The roughness of BMG was far lower than that of steel and slightly lower than that of brass [15]. Han et al. researched the influence of spindle speed, feed rate, and depth of cut on the machined surface of Zr-based BMG. The results showed that the influence of spindle speed on the machined surface of BMG was greater than that of the feed rate and depth of cut, and the greater the spindle speed, the smaller the surface toughness [16]. Chen et al. used a diamond tool and a boron nitride tool to study the cutting performance of Zr_55_Cu_30_Al_10_Ni_5_ BMG. It was found that after ultra-precision machining, the surface of Zr-based BMG showed obvious cutting softening and a nanocrystallization phenomenon, showing poor cutting performance, and it was also difficult to obtain a mirror face with a depth of cut of 0.6 μm [17].

Pd-based BMG has a very strong oxidation resistance, corrosion resistance, and amorphous forming ability, and it is easy to access [18,19,20,21,22,23]. Due to Pd-based BMG having the properties of excellent cutting performance and an easily produced optical mirror surface, this makes it an important material in the field of optics. With the development of new materials and the improvement of cutting tools, the ultra-precision machining technology has become the main means of manufacturing precise parts and optical components [24,25,26]. So far, research on the ultra-precision machining of Pd-based BMG is still very scarce, lacking theoretical and experimental references. In this paper, the effects of cutting parameters on the surface quality of BMG were studied. The oxidation mechanism and inhibition method of BMG were analyzed theoretically, and the effects of tool wear and chip morphology on the surface oxidation were studied. This paper provides an important reference for the research on the ultra-precision machining of BMG, and also provides important theoretical and technical support for the turning and manufacturing of BMG in the optical mold process.

## 2. Experiments and Simulation

### 2.1. Experiments

**Figure 1:**
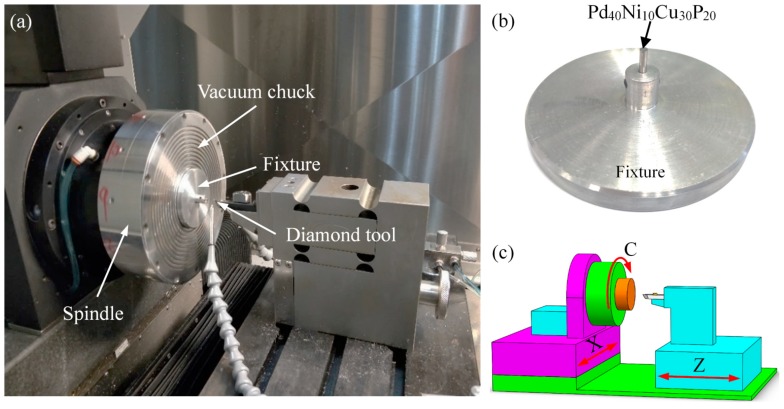
The experimental setup for cutting (**a**), the workpiece (**b**), and a schematic diagram of the 450 UPL ultra-precision lathe (**c**).

The ultra-precision lathe (Nanotech 450 UPL, Moore Nanotechnology Systems, Swanzey, NH, USA) was employed to perform the cutting of rod-shaped Pd_40_Ni_10_Cu_30_P_20_ BMG, as shown Figure 1a. The BMG had a diameter of 2 mm and a length of 16 mm, as shown in Figure 1b. The ultra-precision lathe had three axes, consisting of two linear axes (X and Z axes) and a 360° rotatable axis (C axis), as shown in Figure 1c. A nature diamond tool was employed to perform the cutting, and the tool geometric parameters are given in Table 1. In order to investigate the influence of cutting parameters on the machining performance of Pd-based BMG, the experiments were divided into three groups: group 1, group 2, and group 3. The cutting parameters of the three groups are shown in Table 2. The oil-based lubricant (Isopar H, Exxon Mobil Corporation, Irving, TX, USA) was used during the cutting experiments. The phase structure of the Pd-based BMG on the machined surface was detected using an X-ray diffractometer (XRD) (MiniFlex 600, Rigaku Corporation, Tokyo, Japan) with Cu Kα radiation. The chip morphology and tool wear were measured using a scanning electron microscope (SEM) (ESEM Quanta 450 FEG, Field Electron and Ion Company, Hillsboro, OR, USA). The topography of the machined surface was observed using atomic force microscope (AFM) (MFP-3D Infinity, Oxford Instruments, Abingdon, UK) with a semi-contact mode. The machined surface roughness (*Ra*) was measured using a white light interferometer (Contour GT-X, Bruker, Billerica, MA, USA).

### 2.2. Molecular Dynamics (MD) Model

**Figure 2:**
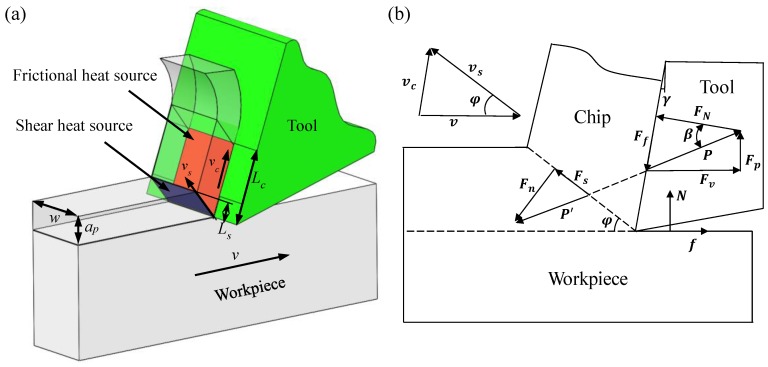
Force components (**a**) and material flows in orthogonal cutting (**b**).

**Figure 3:**
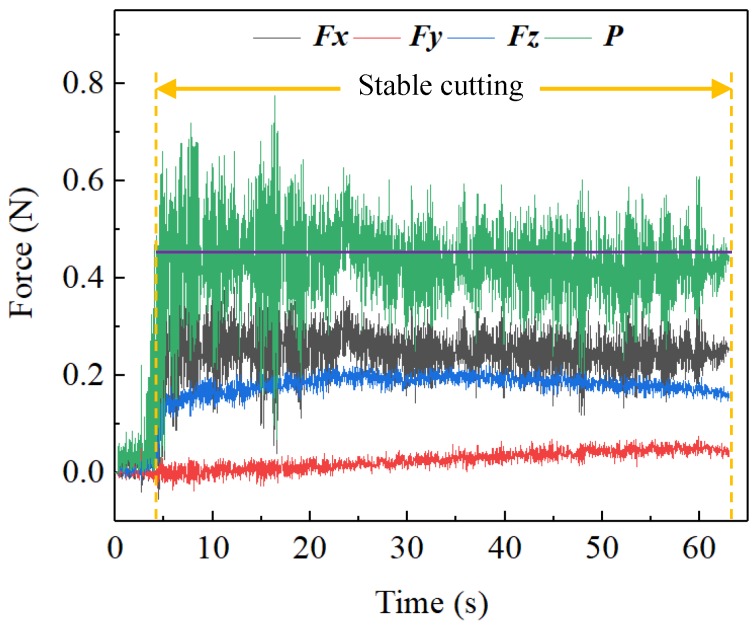
Cutting forces formed in the diamond cutting.

**Figure 4:**
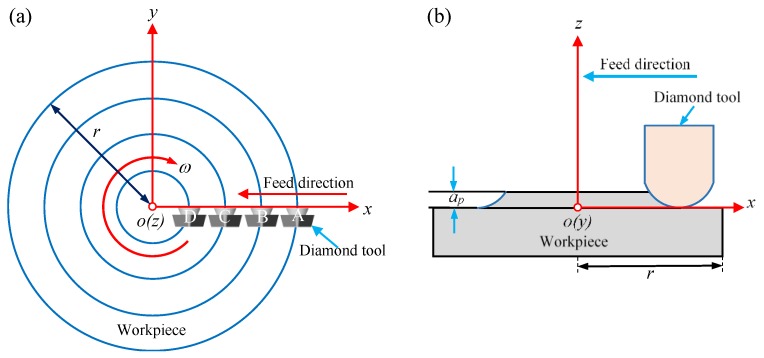
Schematic of the cutting: top view (**a**) and side view (**b**).

The cutting heat mainly comes from two zones [27], as shown in Figure 2. One is the primary deformation zone, where the workpiece material is cut into chips by the tool, which is called a shear heat source, and this causes a sudden increase in the temperature of the chip area near the workpiece and the primary shear plane. The other is the secondary deformation zone, in which the chip is in frictional contact with the rake face of the tool. It is called the friction heat source, which causes severe wear on the rake face of the tool [28]. According to Smith’s definition of orthogonal cutting heat source intensity, the total cutting heat source intensity *Q* is equal to the shear heat source *Q_shear_* of the primary deformation zone and the friction heat source *Q_friction_* of the secondary deformation zone. The cutting heat sources formula is as follows [29,30]:

(1) $$\left\{ \begin{array}{l} Q=Q_{shear}+Q_{friction}\\ Q_{shear}=\frac{F_{s}v_{s}}{L_{s}w}=\frac{Pv\cos\left(\varphi +\beta -\gamma \right)\cos\gamma }{L_{s}w\sin\left(90{}^{\circ}+\gamma -\varphi \right)}\\ Q_{friction}=\frac{F_{f}v_{c}}{L_{c}w}=\frac{Pv\sin\beta \sin\varphi }{L_{c}w\sin\left(90{}^{\circ}+\gamma -\varphi \right)} \end{array}\right.$$

where *F_s_* is the shear force in the primary deformation zone, *v_s_* is the shear velocity in the primary shear plane, *L_s_* is the length of shear band, *w* is the width of cutting area, *F_f_* is the frictional force in the secondary deformation zone, *v_c_* is the chip flow velocity, and *L_c_* is the length of sticking zone and sliding zone. From Equation (1), it is found that both the *Q_shear_* and the *Q_friction_* depend on the cutting resultant force (*P*) and the cutting velocity *v* as the other parameters are constant. It can be observed from the cutting force signal diagram that the *P* was relatively stable throughout the cutting process, as shown in Figure 3, such that the *P* was regarded as a constant, thereby the cutting heat source was only affected by the cutting velocity *v*. In order to study the relationship between the cutting velocity and cutting heat source, four equidistant points A, B, C, and D along the X-axis direction of the workpiece surface were chosen for analysis, as shown in Figure 4.

**Figure 5:**
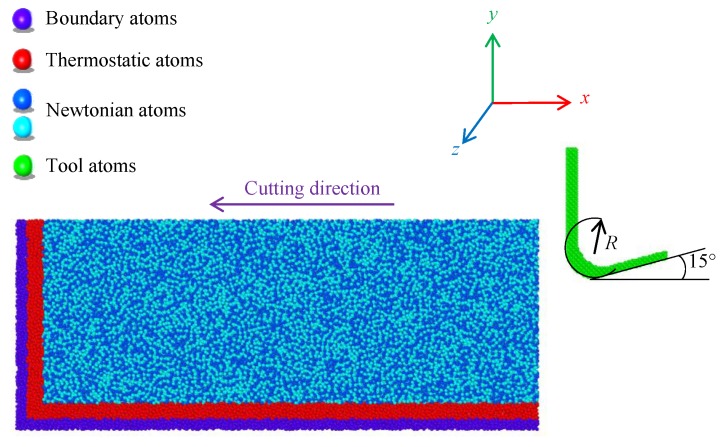
Model of the molecular dynamics simulation.

The MD simulation software LAMMPS (18 Jun 2019 version, Sandia National Laboratories, Albuquerque, NM, USA) [31] was employed for the simulation of the cutting. The cutting model consisted of a diamond tool model and an amorphous workpiece model [32,33,34], as shown in Figure 5. The tool was set to be rigid with the rake angle and the clearance angle of 0° and 15°, respectively, and the tool nose radius was 2 nm. The size of the workpiece was 35 nm × 14 nm × 2.5 nm. It was composed of three kinds of atoms: Newtonian atoms, thermostatic atoms, and boundary atoms. Four layers of boundary atoms at the bottom of the workpiece were used to support the entire system, while six layers of thermostatic atoms above the fixed layer were used to maintain them at a constant temperature of 298 K by rescaling the velocities of the atoms. And others are Newtonian atoms which were used to simulate the movement mode and trajectory of atoms in the cutting process. A periodic boundary was used in the Z direction to reduce the size effect caused by the simulation scale. During the cutting process, the system temperature and energy would change continuously as the cutting progressed. The volume of the workpiece simulation model did not change much under the tool cutting. Therefore, the micro-canonical ensemble (NVE) was selected to simulate the real environment, and the time step was 1 fs. In order to study the effect of cutting velocities on the workpiece temperature, the cutting velocity was set to 20, 40, 60, and 80 m/s along the X-axis direction, and the entire cutting distance was 30 nm. The atomic temperature in the cutting simulation can be obtained by the conversion of kinetic energy (K.E.), as shown in Equation (2):

(2) $$\frac{1}{2}\underset{i}{\sum }m_{i}v_{i}^{2}=\frac{3}{2}Nk_{b}T$$

where *N* is the number of atoms; *v_i_* represents the velocity of the *i*th atom; *k_b_* is the Boltzmann constant, which is equal to 1.3806503 × 10^−23^ J/K; and *T* is the temperature of the atom.

## 3. Results and Discussion

### 3.1. Surface Quality

**Figure 6:**
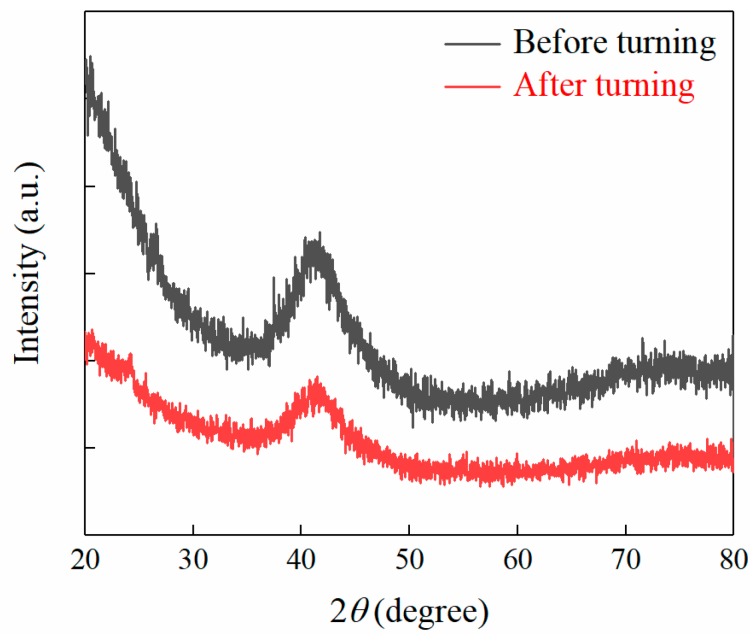
XRD pattern of Pd-based BMG.

In the process of turning, the BMG was easily crystallized, so it was necessary to judge whether the BMG was crystallized. Since the crystallization of BMG did not only cause changes in its structure, but also greatly affected its physical properties, mechanical properties, and machinability, the XRD was first performed on the machined surface, as shown in Figure 6. It can be observed from the XRD pattern that the machined surface of Pd-based BMG had an amorphous structure and exhibited an amorphous diffuse diffraction peak. No crystal phase appeared and it remained amorphous, which means that ultra-precision machining could cut it without changing the amorphous state of BMG. It may be crystallized during cutting, but it did not affect the amorphous state of machined surface after turning due to its good former glass property; therefore, the content is not discussed further here. However, it lay a foundation for the subsequent experiments.

**Figure 7:**
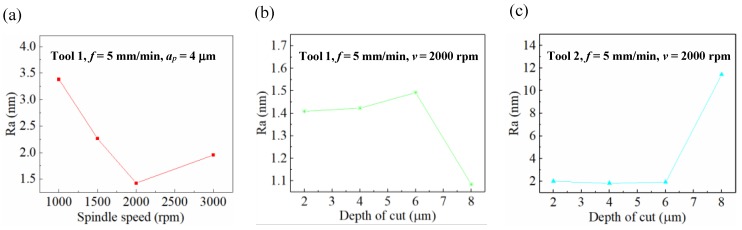
Influence of cutting parameters on roughness of the machined surfaces, at (**a**) Tool 1, *a_p_* = 4 μm and different spindle speed, (**b**) Tool 1, *v* = 2000 rpm and different depth of cut and (**c**) Tool 2, *v* = 2000 rpm and different depth of cut.

The influence of cutting parameters on the surface quality of BMG was studied. Figure 7a shows that the *Ra* changed at the spindle speeds of 1000, 1500, 2000, and 3000 rpm. As the spindle speed increased, the *Ra* decreased first and then increased, and the minimum *Ra* occurred when the spindle speed was 2000 rpm. The reason for this phenomenon was that the BMG, whose temperature was between the glass transition temperature (Tg) and the initial crystallization temperature (Tx), i.e., the supercooled liquid state [35], which had superplasticity and self-healing properties [36], or it was in the true liquid state (melting). This made the BMG originally exhibit brittle hardness at room temperature [2,3,37], and eventually had very good plasticity. When the BMG was at the supercooled liquid state or the true liquid state, with the increase of the spindle speed, the surface temperature of the workpiece increased. Figure 7b shows the changes of the *Ra* at the depth of cut (DOC) of 2, 4, 6, and 8 μm. It can be observed that the *Ra* increased first and then decreased. The best *Ra* occurred at the DOC of 8 μm. When the DOC was less than 6 μm, the temperature generated by the cutting process was smaller than Tg, and the BMG was brittle. Therefore, with the increase of the DOC, the machined surface quality deteriorated and the *Ra* increased; however, when the DOC is greater than 6 μm, the temperature generated by the cutting process exceeded Tg, and the BMG displayed superplasticity, and the *Ra* decreased correspondingly. Figure 7c shows the *Ra* changes with respect to the DOC of 2, 4, 6, and 8 μm, when the tool nose radius was 0.101 mm. It was found that the *Ra* gradually decreased under a DOC of less than 6 μm. However, as the DOC increased to 8 μm, the *Ra* suddenly increased, which is different from common sense.

**Figure 8:**
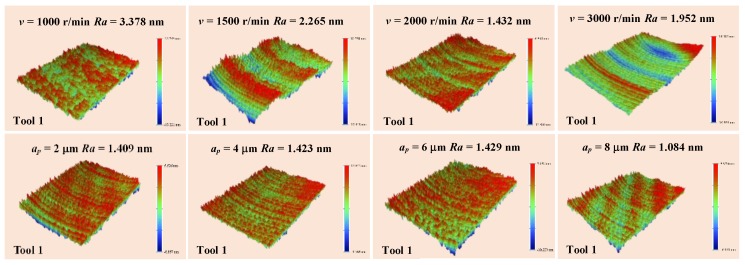
Morphology of the machined surfaces measured using a white light interferometer.

**Figure 9:**
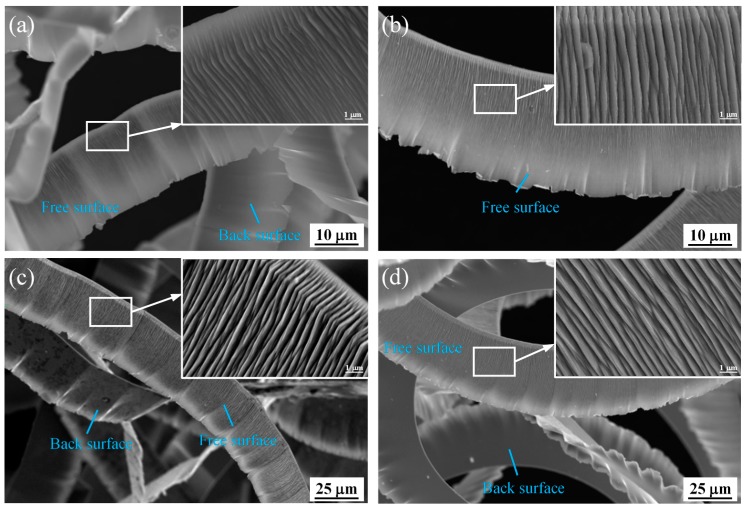
SEM images of chips formed by cutting at a depth of cut of (**a**) *a_p_* = 2 μm, (**b**) *a_p_* = 4 μm, (**c**) *a_p_* = 6 μm, and (**d**) *a_p_* = 8 μm (other parameters: spindle speed of 2000 rpm, feed rate of 5 mm/min).

The superplastic properties of BMG made the machined surface very smooth, as shown in Figure 8, and continuous chips were formed, as shown in Figure 9. As the DOC increased, the free surface wrinkles of the chips increased and the chips still exhibited a continuous band shape. When the cutting temperature of the Pd-based BMG was raised Tg, reaching the supercooled liquid state, the brittle BMG displayed an excellent cutting performance.

**Figure 10:**
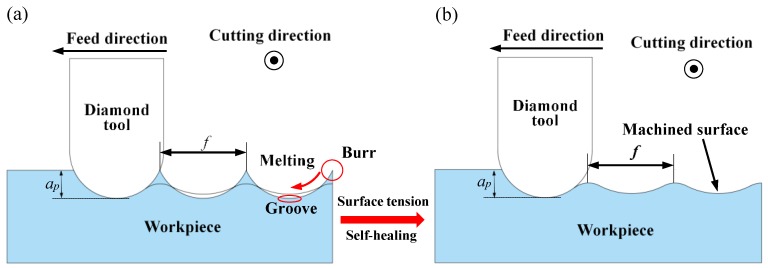
Schematic diagram of the cutting process of BMG, initial state (**a**) and final state (**b**).

**Figure 11:**
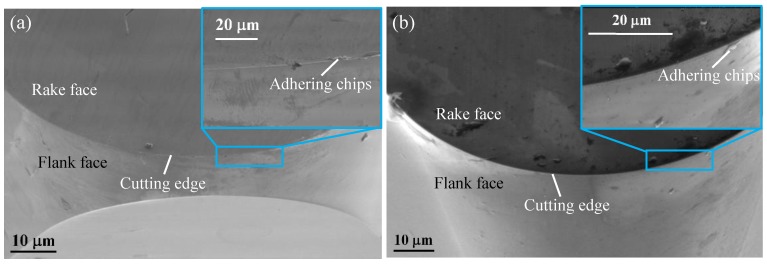
Cutting tool images: Tool 1 micrograph (**a**) and Tool 2 micrograph (**b**).

When the BMG was at the supercooled liquid state or the true liquid state, which had the ability to “self-heal” with the surface tension. When machining ordinary metal materials, such as aluminum and copper, burrs and grooves are usually formed on the surface, as shown in Figure 10a. The BMG was affected by the surface tension like glass, which softened the burr and flowed into the groove, and then exhibited a self-healing ability, thereby improving the surface quality. Moreover, increasing the temperature to reach the Tg during the cutting will caused the structure of the BMG to relax, thereby reducing the fracture toughness and making the BMG easier to cut, finally achieving a better machined surface [2,38,39,40,41,42], as shown in Figure 10b. Therefore, when the DOC was at 4 μm and the spindle speed was below 2000 rpm, as the spindle speed increased, the surface temperature of the workpiece gradually increased. When it exceeded Tg, the surface softened gradually, and improved the cutting performance and decreased the *Ra*, as is shown in Figure 7. When the spindle speed was at 2000 rpm, the self-healing effect was the greatest and the cutting performance was optimal, such that the surface quality was the best and the *Ra* was the smallest. When the spindle speed continued to increase, the surface of the workpiece gradually became sticky. At this time, the tool had an obvious built-up edge, which led to a decrease in cutting performance, as shown in Figure 11.

**Figure 12:**
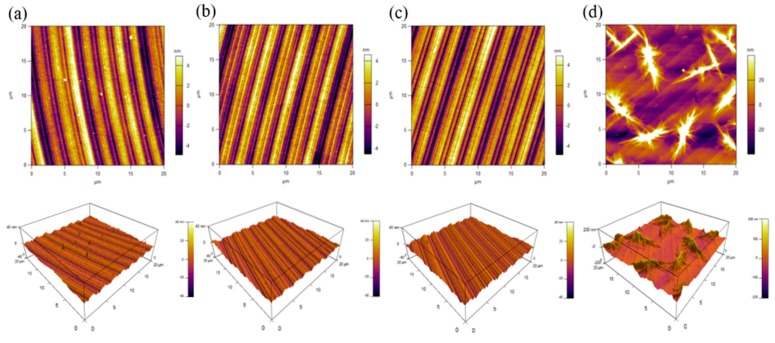
AFM images of the machined surface cut by tool 2 at a depth of cut of (**a**) *a_p_* = 2 μm, (**b**) *a_p_* = 4 μm, (**c**) *a_p_* = 6 μm, and (**d**) *a_p_* = 8 μm (other parameters: spindle speed of 2000 rpm, feed rate of 5 mm/min).

In order to investigate the abnormal phenomena on the *Ra* and the origins, the AFM was used to characterize the topography of the BMG surface. When the DOC was at 2, 4, or 6 μm, the machined surface only had obvious tool marks, as shown in Figure 12a, b and c. When the DOC was at 8 μm, some irregular micro/nanostructures were found on the machined surface, as shown in Figure 12d. These structures resulted in an increase in the *Ra* of BMG and a consequent deterioration in quality.

### 3.2. Surface Oxidation

**Figure 13:**
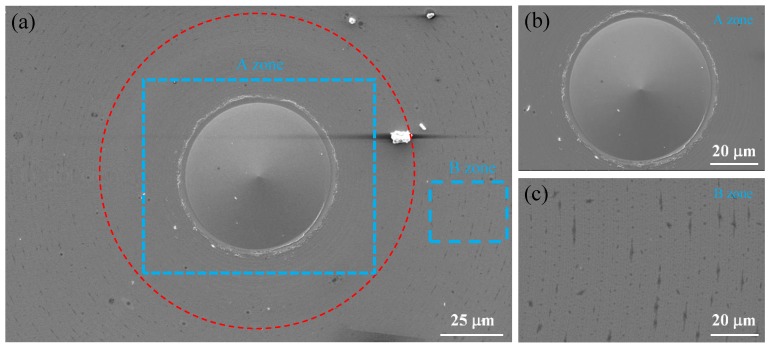
The surface morphology of the whole workpiece (**a**), the center area (**b**) and outer circle area (**c**).

In order to study the distribution of the irregular micro/nanostructures generated on the surface of Pd-based BMG, the surface morphology was measured using the SEM. From Figure 13a, the irregular micro/nanostructures could be observed as being distributed only on the outer circular region of the machined surface, which were along the trace of the tool marks, as seen in the enlarged image in Figure 13c. Meanwhile the center area of the machined surface was relatively clean and had no irregular micro/nanostructure exists, as shown in Figure 13b.

**Figure 14:**
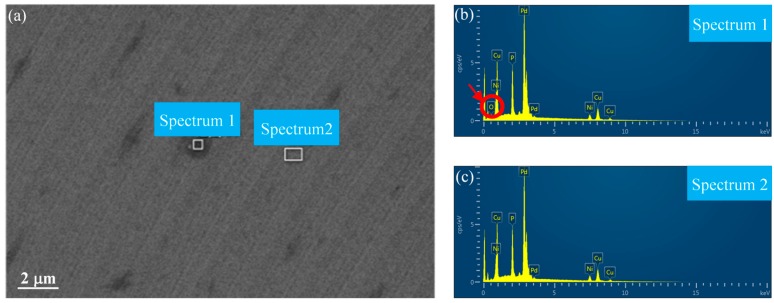
EDS result of the workpiece surface (**a**) with the associated spectrum 1 (**b**) and spectrum 2 (**c**).

In order to study the origins of the irregular micro/nanostructures on the surface of the Pd-based BMG, the composition of the micro/nanostructures was measured using an energy dispersive X-ray spectrometer (EDS). As shown in Figure 14a, the energy spectrum analysis was performed on the micro/nanostructure region (spectrum 1) and the clear region (spectrum 2) on the workpiece surface. Oxygen elements were found in spectrum 1, as shown in Figure 14b, while spectrum 2 did not display the presence of oxygen, as shown in Figure 14c. Therefore, irregular micro/nanostructures were caused by oxidation, and the oxidation was only distributed along the tool mark on outer circular region of the machined surface.

**Figure 15:**
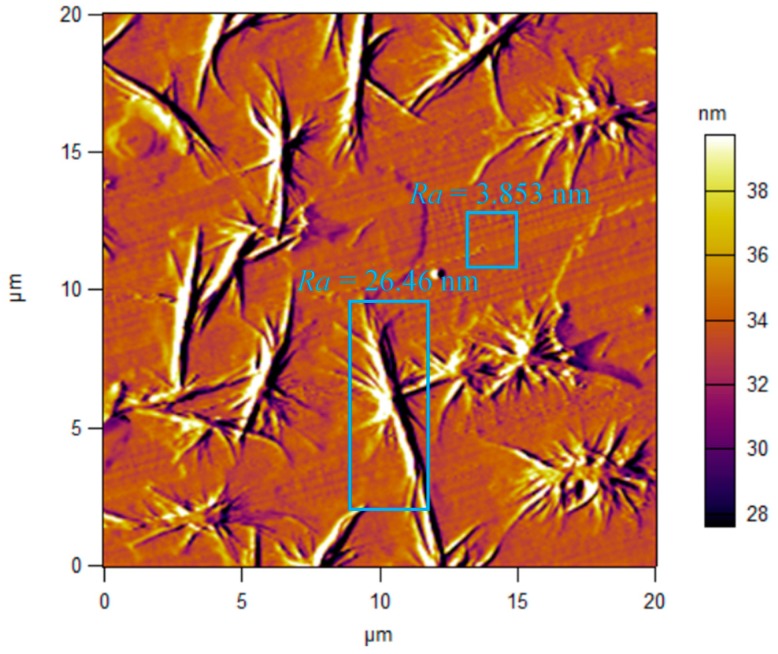
Irregular micro/nanostructures formed on the machined surface.

It can be observed from Figure 15 that the *Ra* of the irregular micro/nanostructures produced by the oxidation of the Pd-based BMG was 26 nm, while the *Ra* of the smooth surface was only 3.8 nm, proving that the oxidation seriously affected the surface quality of the BMG and led to the increase of *Ra*. BMG is a material with a high amorphous forming ability and extremely high oxidation resistance. However, when the temperature of the cutting zone is too high and is above Tg for a long time, BMG is susceptible to oxidation. The case of BMG entering a true liquid state (melting) can be excluded, because the machined surface in this case should be completely smooth without any tool marks; however, notable tool marks were found in Figure 15.

### 3.3. MD Simulation Results

**Figure 16:**
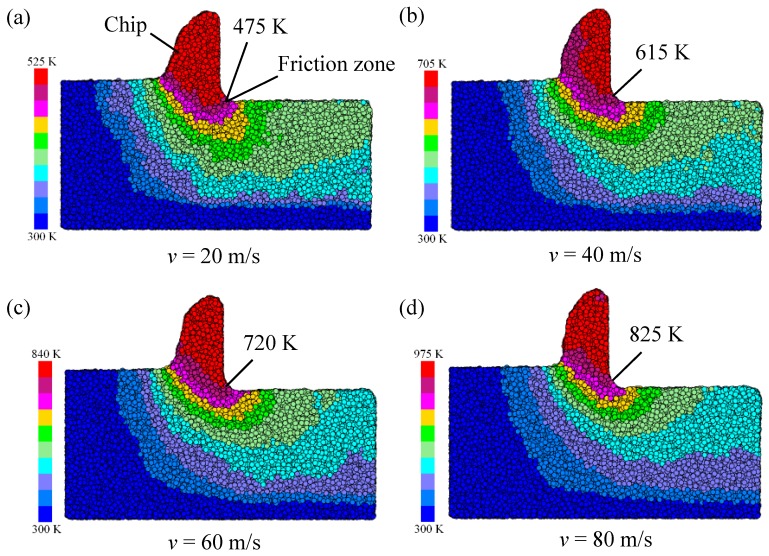
Evolution of the temperature distribution in the deformation zone with different cutting speeds, (**a**) *v* = 20 m/s, (**b**) *v* = 40 m/s, (**c**) *v* = 60 m/s and (**d**) *v* = 80 m/s.

Figure 16 shows the temperature distribution of the workpiece. Taking the chip action area as the center, the highest temperature of the workpiece was generated at the chip, and there was also a high-temperature zone in the friction zone of the tool flank. The temperature of the subsurface and the machined surface of the workpiece was also relatively high, the temperature inside the workpiece near the boundary was low, and the temperature gradient was large. The highest temperature at the chip was due to the maximum deformation of the chip during the cutting process. The atomic energy in this zone had the largest deformation energy, and the temperature was related to the energy of the atom. The friction zone of the workpiece was severely compressed by the tool. The deformation of the workpiece atom was relatively large, and the atom in this zone had a large amount of energy, such that the temperature in this zone was also high. When the cutting velocity was at 20 m/s, the temperature of the friction zone reached 475 K; therefore, the BMG temperature in the cutting zone was below the glass transition temperature (Tg = 670 K). When the cutting velocity was at 20–80 m/s, the BMG temperature of the friction zone reached 720 K, and the BMG temperature in the cutting zone was between the glass transition temperature (Tg = 670K) and the initial crystallization temperature (Tx = 720K). When the cutting velocity was increased to 80 m/s, the temperature in the friction zone of the flank reached 825 K. At this time, the workpiece exceeded Tx, and BMG began to be crystallized.

**Figure 17:**
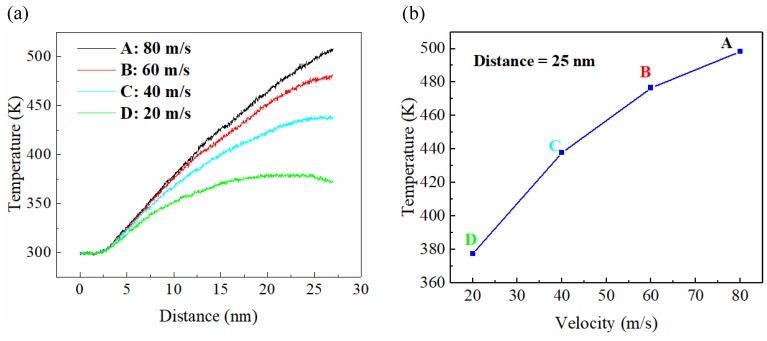
Temperature of the BMG with different cutting speed as cutting distance increases (**a**) and diagram of the BMG temperature vs. cutting speed at a cutting distance of 25 nm (**b**).

Figure 17a shows the curve of the workpiece temperature change with cutting distance at different cutting velocities, while the workpiece temperature with respect to different cutting velocities at a cutting distance of 25 nm is shown in Figure 17b. The cutting velocity had a great influence on the workpiece temperature, where it was found that the temperature of the workpiece rose with the increase of tool cutting velocity, and the increase of the cutting velocity also caused the larger slope of the curve, indicating a quicker temperature increase. In the initial phase, the temperature remained constant because the tool had not touched the workpiece and the temperature rose rapidly when the tool touched the workpiece. The increase in cutting velocity increased the heat generated in the cutting area. If the heat was not diffused in time, the temperature in the cutting area exceeded Tg, and the cutting area became viscous, which caused additional friction between the tool and the workpiece. The surface of the workpiece was more intense, which caused the workpiece temperature to continue to rise, and if the temperature was above Tg for a long time, a surface oxide was generated.

## 4. Conclusions

The cutting mechanism and characteristics of Pd_40_Ni_10_Cu_30_P_20_ BMG in SPDT was studied both theoretically and experimentally. The surface generation and oxidation characteristics at different cutting parameters were investigated. Furthermore, an MD simulation of the oxidation characteristics was carried out. Specific conclusions are as follows:

(1) Pd-based BMG had an excellent cutting performance and the machined surface quality could achieve an optical mirror level with a small DOC. However, when the DOC exceeded a certain DOC (around 8 μm here), some irregular micro/nanostructures were formed along the tool path on the outer circular region of the machined surface and seriously affected the machined surface quality.
(2) When the temperature of the Pd-based BMG cutting area was between Tg and Tx, BMG was in the supercooled liquid state, which has superplastic properties, whereby the tool mark was flattened under the effect of surface tension, and therefore improved the quality of the machined surface.
(3) The superplastic properties of the BMG provided the material with a high viscosity, which caused the tool to produce a built-up edge phenomenon, affecting the machined surface quality, increasing the cutting heat, and further causing the oxidation of the machined surface.

This paper provides a deep insight into the diamond-cutting mechanism of BMG materials, as well as providing theoretical and technical support for the machining and manufacturing of BMG optical molds.

## Figures and Tables

**Table 1:** Geometric parameters of the tool employed in the experiment.

Tool Parameters	Tool 1	Tool 2
Tool materials	Nature diamond	
Tool rake angle (α)	0°	
Tool clearance angle (β)	15°	
Tool nose radius (*R*)	0.502 mm	0.101 mm

**Table 2:** Cutting parameters for the diamond turning of Pd-based bulk metallic glass (BMG).

Items	Feed Rate (*f*) (mm/min)	Tool Nose Radius (*R*) (mm)	Spindle Speed (*v*) (rpm)	Depth of Cut (*a_p_*) (μm)
Group 1	5	0.502	1000, 1500, 2000, 3000	4
Group 2	5	0.502	2000	2, 4, 6, 8
Group 3	5	0.101	2000	2, 4, 6, 8
